# Prospective measures of aging for Central and South America

**DOI:** 10.1371/journal.pone.0236280

**Published:** 2020-07-24

**Authors:** Stuart Gietel-Basten, Silvia E. Giorguli Saucedo, Sergei Scherbov

**Affiliations:** 1 Division of Social Science, The Hong Kong University of Science and Technology, Clear Water Bay, Kowloon, Hong Kong SAR, People’s Republic of China; 2 Division of Public Policy, The Hong Kong University of Science and Technology, Clear Water Bay, Kowloon, Hong Kong SAR, People’s Republic of China; 3 El Colegio de México, México City, México; 4 World Population Program, International Institute of Applied Systems Analysis, Laxenburg, Austria; 5 International Laboratory of Demography and Human Capital, Russian Presidential Academy of National Economy and Public Administration, Moscow, Russian Federation; Universidade Federal de Minas Gerais, BRAZIL

## Abstract

By conventional measures, it is often remarked that Central and South America is one of the fastest aging geographic regions in the world. In recent years, however, scholars have sought to problematize the orthodox measures and concepts employed in the aging literature. By not taking dynamic changes in life expectancy into account, measures which hold chronological age constant (e.g. defining a boundary to old age at 60 or 65) represent a very narrow view of population aging. Furthermore, such constant measures may misrepresent differences between territories when performing a comparative analysis. *Prospective* measures based on the number of years until death present an alternative approach which can adapt to dynamic changes in life expectancy and differences over time and space. The objective of this paper, then, is to apply the new ‘prospective’ measures of aging to the territories of Central and South America. We calculate prospective median age; an alternative old-age threshold based on the age at which remaining life expectancy is 15 years, and calculate prospective old-age dependency ratio for 1950–2100 using estimated and projected life tables from the latest iteration of the UN’s *World Population Prospects*. These new measures present a very different view of aging in Central and South America. While there are significant differences across countries, the pace and scale of aging are considerably slower and diminished when compared to standard, orthodox measures based on fixed chronological ages. Applying these new measures can not only serve to present a more realistic view of aging which maps onto demographic reality but can also serve to reconceptualize and reframe the issue as something which is far more manageable (e.g. through institutional reform) than is often perceived to be.

## Introduction

According to a recent study, ‘Latin America and the Caribbean [LAC] is one of the fastest aging geographic regions in the developing world’ [1]. As well as having experienced a rapid demographic change in terms of fertility and mortality, it is further argued that the ‘weakening of familial norms and values, and radical reforms of publicly funded safety nets, is rapidly eroding the foundation of traditional support for the elderly’ [2]. As in other parts of the world, both parametric and paradigmatic reforms to pension and other social welfare systems [3–6] are being proposed in order to ‘maintain fiscal balance and sustain economic prosperity’ [7]. Elsewhere, extensive reforms to healthcare and insurance systems [4,8] have been implemented, as well as more proactive approaches such as ‘active ageing’ programs [9,10]. In common with other aspects of social and economic change, inequalities also play an important role in shaping the lived experiences of older persons and, as a consequence, shape the necessary policy responses [11,12].

But, the measurement and definition of ‘old age’ is also crucial to determining the nature and scale of the challenge as well as how we communicate the ongoing changes in the population age structure. Standard definitions of ‘old age’ are 60 and 65 years old–in part a legacy from the link to retirement ages and pension entitlement [13]. These boundaries to old age are reflected in many studies of Central and South America which describe the scale of aging using traditional measures based upon the threshold of either 60 [2,14–16] or 65 [1,17]. These traditional measures include the median age of the population, the percentage of the total population aged over 60/65 and, strongly related to this, the ‘old age dependency ratio’ (*OADR*) which is the number of people aged over 60/65 divided by the ‘working-age population’ (usually a lower bound of 18 or 20). For example, 60 was set as the ‘boundary’ to old age’ (and 15 as the lower threshold for the ‘working population’) in a chapter by Saad in a 2010 World Bank Report entitled *Population Aging*: *Is Latin America Ready*? [15]. A more recent study by the IMF, meanwhile, placed the ‘boundary to old age’ at 65 [18]. The study by Saad [15] referred to the dependency ratio as ‘a valuable indicator of the potential effects of demographic changes on socioeconomic development’ on the basis that ‘a high proportion of economically dependent persons in the population (children and older persons, in general) usually constrain economic growth, because a significant portion of resources [are] allocated to attend to their needs’. ‘In contrast,’ Saad continues, ‘a large share of working-age people can boost economic growth, since a larger proportion of workers and a lower level of spending on dependent persons tends to accelerate the accumulation of capital’ [15]. Using this measure for projections through to 2050, the study by Saad [15] indeed finds ‘a strong upward trend’ in aging among certain countries. Saad reflected on these changes in the *OADR* by noting that ‘sooner for some countries and later for others, the advantage of a favorable ratio between the working-age population and the dependent-age population will disappear as the share of older persons steadily increases’. ‘If this phase is reached in adverse economic conditions, with little or no economic growth or accumulated savings,’ the study continues, ‘the burden placed by the dependent older population on the economically active population will require huge transfers of resources from the latter to the former, which might create not only intergenerational conflict, but also solvency problems that could jeopardize the financing of key systems, such as health care and social security.’ Without doubt, the standard narrative is that in Central and South America–albeit with strong regional differences–there is forecast to be a steep increase in the standard measure of ageing in the near future, namely the median age, percentage of population aged above 60/65 and the *OADR*. This, in turn, shapes the narrative of the perceived pace, scale and severity of population aging in Central and South America [12] –and beyond.

Although exceptions exist [19], these standard measures tend to be used in an unthinking manner; taken as a given and part of the common demographic lexicon. In recent years, however, these standard measures have been subject to increasing scrutiny and critique. A focus on healthy (or specific disease-free) life expectancy, for example, has helped move beyond the binary of life and death to more sharply focus on changing health issues and needs [20]. However, a more conceptual shift has developed in terms of how we measure or determine ‘old age’ at all. In particular, an argument has been developing that we have become ‘too dependent on conventional dependency ratios’ (based on a threshold of 60 or 65) [19].

**Fig 1:**
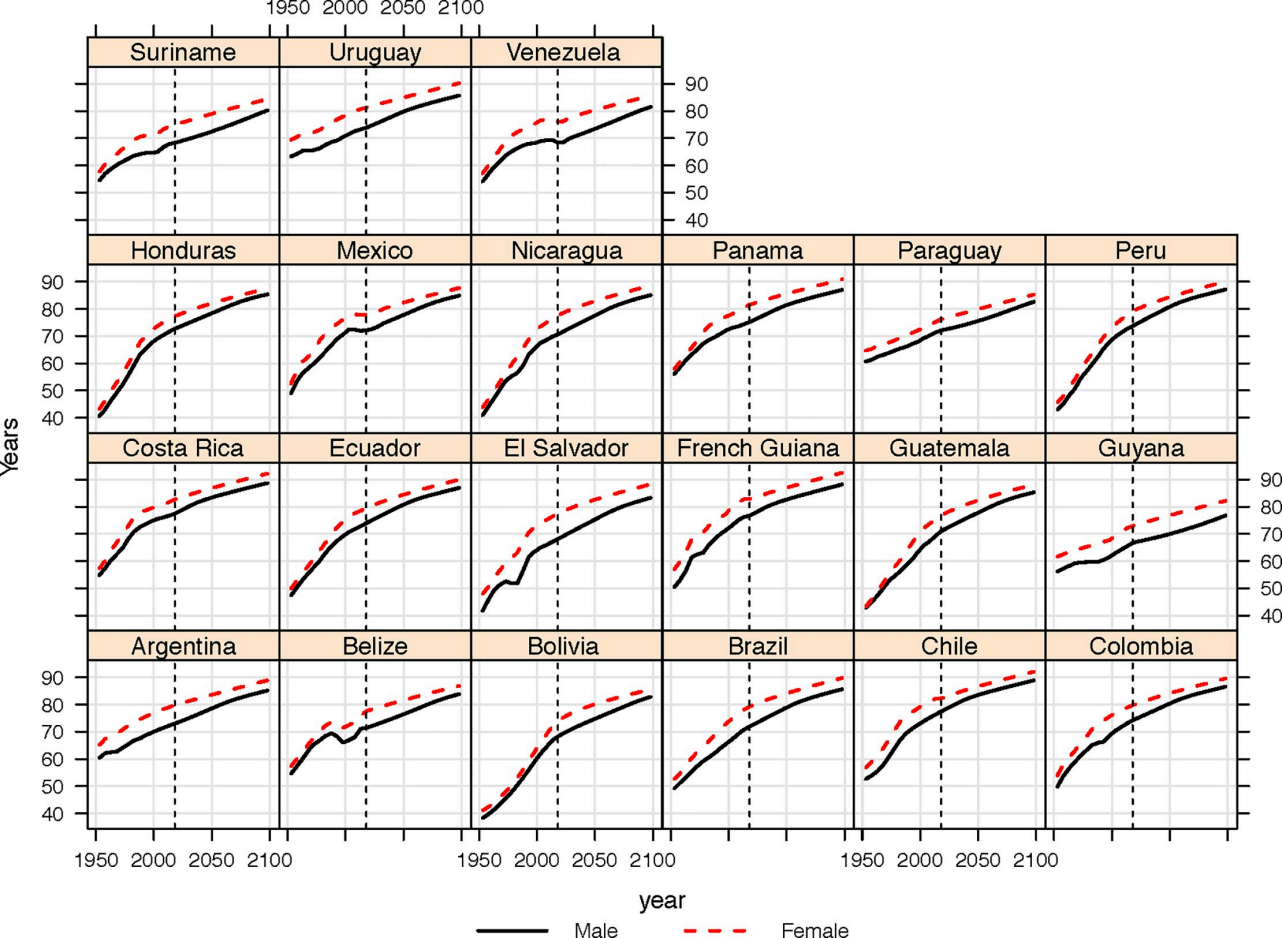
Past and projected changes in life expectancy at birth, males and females, 1950–2100, Central and South American countries. Source: [28].

Biologically, of course, there is no dramatic change in a person’s body which occurs on their 65th birthday–or, indeed, within the 65th year of life. More importantly, as Fig 1 demonstrates, Central and South American countries have also undergone a revolution in life expectancy. This phenomenon and the underlying drivers are, of course, well known [21–23] –although there is still significant divergence between the countries today [24]. Apart from the general shift through the processes of the epidemiological transition [25–27] and burden of disease which are common with other countries, particular country specific trends in mortality and morbidity can also be observed, whether these are related to homicide [24], diabetes [20] or other pathways of health at older ages [7].

We can, however, extend this well-trodden discussion by introducing the concept of measuring aging *prospectively*. Elaborated in a number of articles by Warren Sanderson and Sergei Scherbov [29–31], this concept states that while two people in two different places or times with the same *chronological* age may have relatively little in common, two such people with the same *remaining* age may share more similarities. Extending this, if we ‘fix’ remaining life expectancy over time–or space–we are more likely to be comparing like-with-like. As such, rather than determining the boundary to old age as 65 and holding this constant across all countries for all times, we can fix a given number of years remaining life expectancy (RLE) and show how the boundary to old age changes over time which takes into account dynamic changes in life expectancy (and, also, health and other characteristics).

Finally, by changing the boundary to old age it is therefore logical that one can recalculate *OADR* upon the principle of measuring age prospectively. This so-called *prospective old-age dependency ratio* (*POADR*) represents a simple, intuitive alternative to *OADR*; it has the primary benefit of taking into account dynamic changes in life expectancy. In common with the *OADR*, the lower threshold of the *POADR* is set at 20 years. However, the upper bound is set as the age at which RLE is equal to 15 years. This figure has been set as it is broadly coincides with the RLE at pensionable age at a time when many pension schemes were introduced in the mid-twentieth century (albeit in other parts of the world). Other boundaries could, of course, be set. The *POADR* is, to a degree, divorced from the prevailing institutional framework; but so too is the *OADR*. Rather, it simply represents an alternative way to conceptualize the changes in aging in a holistic manner; and one which, from a policy perspective, shows how policies such as active aging or age-friendly cities which are designed for ‘older people’ need to be more circumspect when considering the target audience based on chronological age alone. Based on this criteria, the *POADR* has been calculated for various world regions including Oceania [32], East Asia [33–35], Southeast Asia [36] as well as numerous other territories [31,37] and even at the sub-national level [38].

Our objective in this paper is to present a set of alternative measures of aging for Central and South American countries based upon the principle of prospective aging. We calculate the age at which RLE = 15 in Central and South American countries as well as the *POADR*, and compare this to the standard *OADR*.

## Materials and methods

For the calculation of the old-age threshold and *POADR*, various studies by Scherbov and Sanderson suggest setting the boundary at 15 years because this was the RLE at 65 in many European and North American settings when pension systems were being rolled out in the mid-twentieth [e.g. 30,31]. A further principle of this prospective approach is that healthcare costs are more strongly related to proximity to death than chronological age [39]. The institutional and demographic experience of Central and South America was very different to Europe and North America over much of the twentieth-century; however we keep an RLE of 15 years as an instructive boundary which can relate to a ‘standard’ from elsewhere. We calculate the age at which RLE = 15 as well as the *POADR* for all territories in the region. We then compare these with the calculated *OADR*. Of course, significant data quality issues undoubtedly exist across the dataset; especially in terms of the reconstruction of historical life tables. Quinquennial life tables are published by the UN and the latest Revision of such tables are used in this exercise(UNPD 2019). Initial transformation is required to determine the mid‐interval average, followed by spline interpolation to get the exact time data required for this exercise.

We use national life tables from the 2019 *World Population Prospects* published by the United Nations. Firstly, we will calculate the median age from the observed age distribution and *prospective median age* (which standardizes the median age for changes in life expectancy) [40]. Median age is simply the age that divides a population into two numerically equal groups. The prospective median age, meanwhile, is the age in the reference life table where the remaining life expectancy is the same as at the median age in the specified year. This is inherently hypothetical. For our exercise here we use 2013 as the reference life table.

In formal terms, prospective median age in country *j* in year *t* is derived from the equation:

$${pma}_{j,t}=e_{j,2013}^{-1}[e_{j,t}({ma}_{j,t})]$$

where *pma*_*j*,*t*_ is the prospective median age in country *j* in year *t*, *ma*_*j*,*t*_ is the median age in country *j* in year *t*, *e*_*j*,*t*_(*ma*_*j*,*t*_) is the life expectancy in the life table for country *j* in year *t* at the median age of the population in that year, and $e_{j,2013}^{-1}[e_{j,t}({ma}_{j,t})]$ is the age in the country’s life table of 2013, where remaining life expectancy is the same as the median age in year *t*.

The old age threshold used in the computation of the prospective proportion of the population who are old and the prospective old age dependency ratio is derived from the equation:

$${OAT}_{j,t}=e_{j,t}^{-1}(15)$$

Where *OAT*_*j*,*t*_ is the old age threshold in country *j* in year *t*, and $e_{j,t}^{-1}(15)$ is the age in the life table for country *j* in year *t* where remaining life expectancy is equal to 15 years.

Having calculated this, we can therefore calculate and compare *OADR* and *POADR* as follows for country *j* in year *t*.

$${OADR}_{j,t}=\frac{{population\;aged\geq 65}_{j,t}}{{population\;aged>20\;and<65}_{j,t}}$$

$${POADR}_{j,t}=\frac{{population\;aged\geq OAT}_{j,t}}{{population\;aged>20\;and<OAT}_{j,t}}$$

The results are presented in tabular and graphical form.

## Results

**Fig 2:**
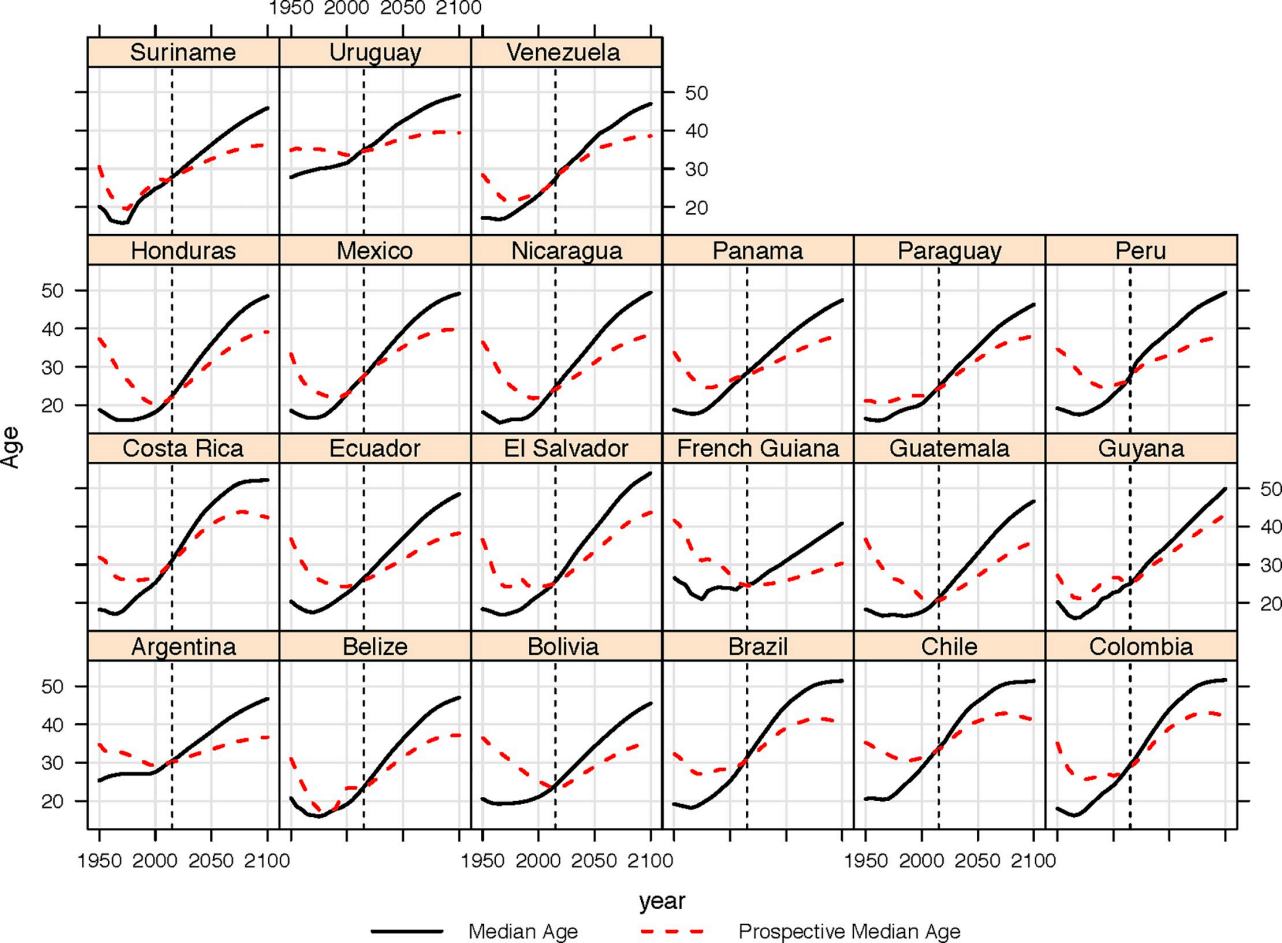
Past and projected changes in median age and prospective median age, 1950–2100, Central and South American Countries, estimates to left of dotted line, projections (using medium variant) right of dotted line. Source: [28].

An overview of the exact figures generated by the exercise can be found in SI1. Fig 2 compares the median age with the *prospective median age* [*pma*]. Because of ongoing improvements in mortality, the increase in the *prospective median age* is forecast to be consistently lower than the median age. While there is a range of differences between countries, we can see that this alternative approach based upon prospective measures serves to rewrite the narrative on the pace of aging in the past (as countries have been effectively getting younger) and also over the next decades.

**Fig 3:**
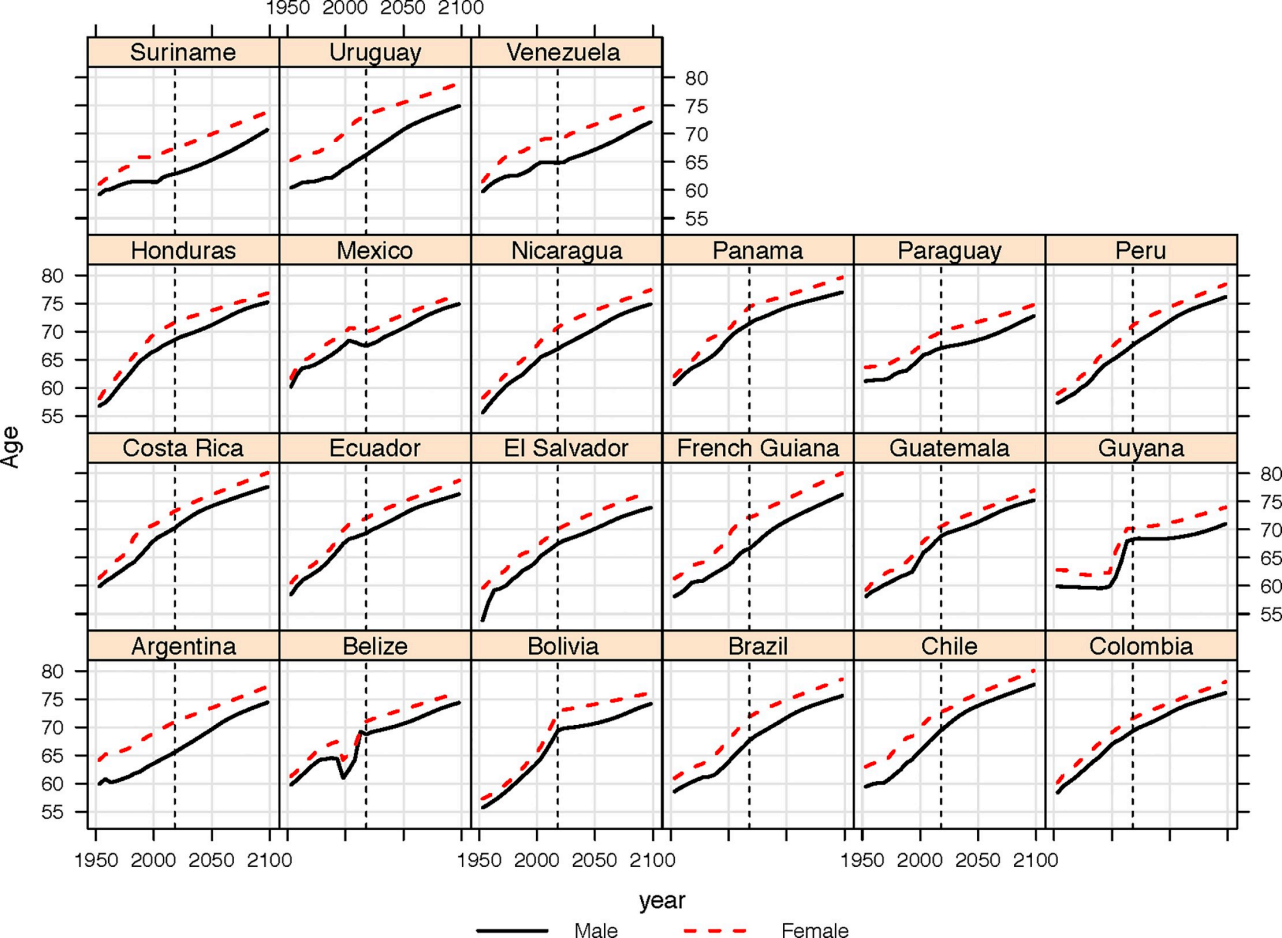
Past and projected changes in the old-age threshold (*OAT*, i.e. age where RLE = 15), 1950–2100, Central and South American Countries, estimates to left of the dotted line, projections (using medium variant) right of the dotted line. Source: [28].

Fig 3 represents the changes in the old-age threshold (*OAT*)–given as the age at which RLE = 15 –in Central and South American territories. Compared to a static threshold of 65, all territories see an increase over the period 1950 to today; and a further increase in the future. In 1980, for males and females taken together, the *OAT* was lower than 65 in 15 of the 21 countries. In Guyana and Bolivia, for example, the *OAT* was 60.8 and 61.2 respectively. Compared, then, to a threshold of 65, it could be argued that these countries were underestimating aging at that time. By 2020, the male and female *OAT* was greater than 65 in all countries. Again, however, we see a sizable range—from 65.8 in Suriname up to 73.6 in Panama. By 2050 it is estimated that the gap between the ‘standard’ indicator of ‘old age’ (i.e. 65) and the *OAT* will be greater than ten years in Chile, Costa Rica and Panama.

**Fig 4:**
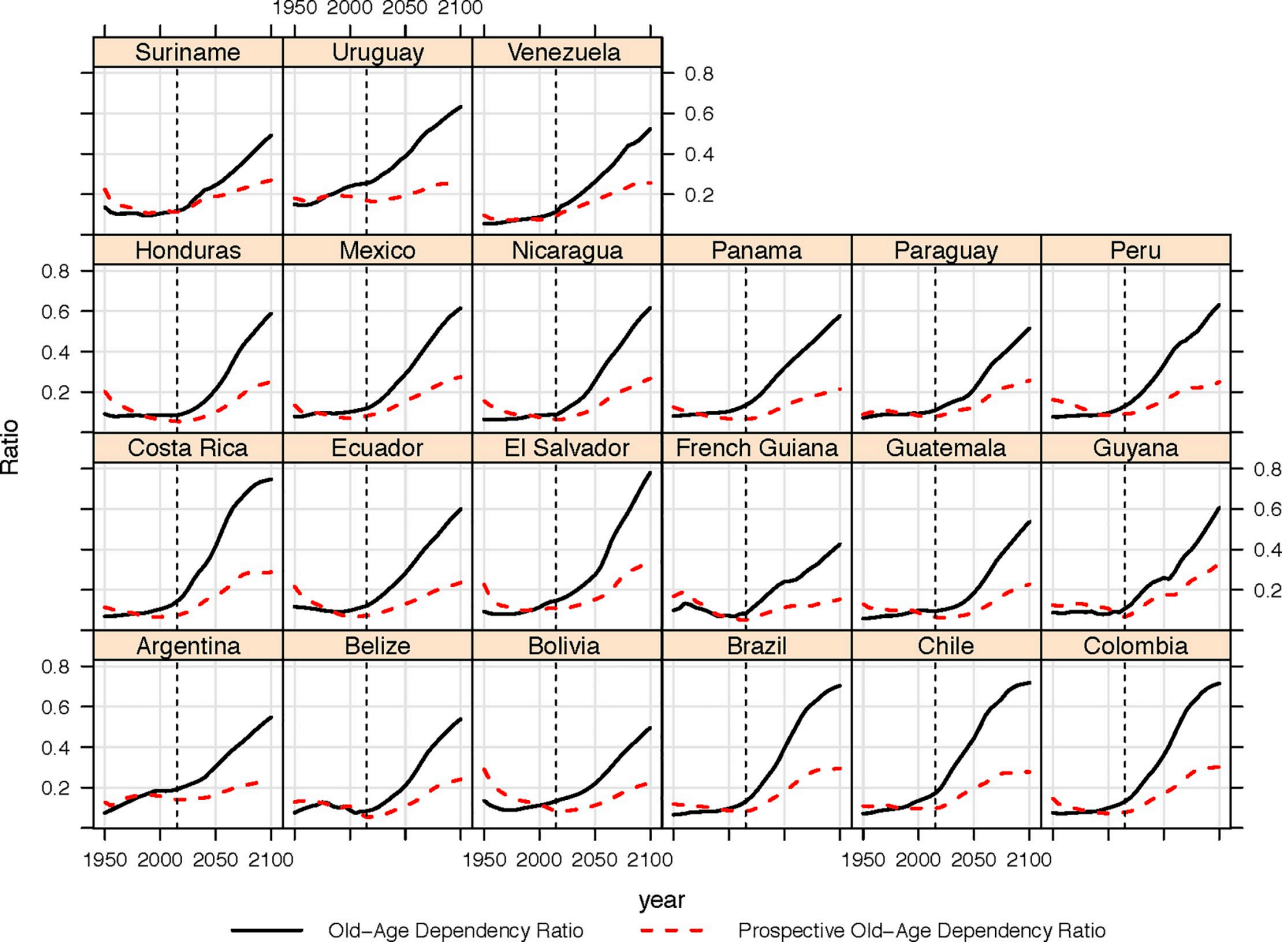
Past and projected changes in the *OADR* and *POADR*, 1950–2100, Central and South American Countries, estimates to left of dotted line, projections (using medium variant) right of dotted line. Source: [28].

Fig 4, then, translates this new, dynamic boundary to old age into a prospective old-age dependency ratio. In every country, the projected *POADR* is lower than the *OADR*. There is, however, a high degree of heterogeneity across the region. Territories characterized by lower fertility and longer life expectancy today (and hence more rapid aging) report the greatest difference between *POADR* and *OADR*. In 2020, such examples include Chile, Uruguay, and Costa Rica. However, in other settings characterized by higher fertility and/or mortality (and slower aging), the difference between *POADR* and *OADR* is much smaller. This is the case in Suriname, Belize and Venezuela, for example. By 2050, the gap between the *OADR* and *POADR* has widened across almost all countries, in some cases very significantly. Consider the case of Chile, for example. In 1980, the *POADR* and *OADR* were both 0.1. By 2050, however, the *OADR* is forecast to be 0.45 while the *POADR* is 0.2. As such Chile, which is often used as an example of a very rapidly aging country with numerous associated challenges (e.g. the pension system), could be said to be aging much more slowly if we use an alternative measurement. Indeed, by 2050 the *POADR* is less than 50% of the *OADR* is 11 of the 21 countries under analysis here, with the greatest difference being Costa Rica, where the *POADR* is 40.4% of the *OADR*. As one might expect, smaller gaps between *OADR* and *POADR* for 2050 are found in settings with higher fertility and lower life expectancy, such as Suriname, Guyana, and Venezuela.

## Discussion

It is frequently said that Central and South American countries have started a ‘new demographic era’ defined mainly by a ‘massive ageing process’ [41]. Without doubt, the standard measures of aging support these kinds of statements. On the other hand, as we have shown above, when we apply the prospective measures of aging proposed elsewhere to the situation in Central and South America, then the changes in population structure are not nearly so ‘massive’. In other words, our results demonstrate that there can be more than one ‘narrative’ when presenting the circumstances of population aging in Central and Latin America.

Determining the most appropriate means of ‘measuring’ aging requires a more precise definition of precisely which aspect of aging is being considered. For some issues, dependency rates based on chronological age boundaries are, perhaps, most useful. This could be the case for when there is a ‘hard change’ at a given age, such as entitlement for a pension, or eligibility for retirement. Indeed, it is in these institutional changes that the roots of the original demarcation of ‘old age’ used in orthodox dependency ratios can be found. These ‘support ratios’ are useful to gain a snapshot of the sustainability (or lack thereof) of existing institutional systems [15]. Yet, even under these circumstances a ‘catch-all’ boundary of 60 or 65 may obscure as much as it shows. Of the 20 Central and South American countries covered by the *Social Security Programs Throughout the World Survey*, only three countries (Costa Rica, Guyana and Peru) set a universal statutory pensionable age at 65 for both men and women [42]. Women in Colombia and Panama are eligible for their pension at age 57, for instance. Again, of course, when we consider labor force participation and other parameters of the social security systems in place across the region we again see the futility of using age 65 and above as a true means of gauging the nature and extent of the so-called ‘aging crisis’.

While perhaps less intuitive than chronological measures, prospective measures of aging arguably offer a more holistic view of the changing population age structure which is divorced from the existing institutional framework (e.g. of pensionable age). In the sense of being delinked from ‘ages of entitlement’, it is neither ‘better’ or ‘worse’ than the classic *OADR* or ‘support ratios’ [43,44]; rather just simply ‘different’. One benefit of using a prospective approach to deliver a more holistic view of aging is that it better takes into account dynamic changes in life expectancy within territories, as well as cross-national heterogeneity. This is important to compare the past, present and future in a given country; but also to better compare between countries. The health status (and life expectancy) of a 65-year old man in Guyana in 1950 is likely to be very different from the health status of a 65-year old man in Guyana in 2050; let alone a 65-year old man in Costa Rica in 2050. Yet all three are considered equally ‘old’ under the standard *OADR* measurements. In fact, the 65-year old men in Guyana in 1950 and Costa Rica in 2050 would have respective remaining life expectancies of 11.9 and 22.4 years [28]. In a sense, then, prospective measures show us very clearly the mismatch between prevailing social institutions and the demographic dynamic. Public decisions around the health system, pensions, Conditional Cash Transfers (CCT) to the elderly or the provision of care have not efficiently incorporated the past and projected trends in health, life expectancy, and age structure. In addition, much of the public discourse surrounding aging generally and the criteria regarding the retirement age or the access to benefits for the elderly are based on a common approach which uses age 60 or 65 as a threshold. As a result, even though population aging is acknowledged to be ‘the single greatest achievement of mankind’ [45], the narrative around the aging process, especially regarding the costs for the health system and of non-contributive pensions, is very pessimistic and points more towards a political stalemate.

Within this scenario, and for a more efficient planning of policies around ageing, the *POADR* and the use of a dynamic old-age threshold based on RLE offers a different perspective. For example, the *POADR* shows a slower aging process and invites policymakers and other stakeholders to analyze and consider labor force participation beyond age 65. This information is relevant in the current discussion regarding the reforms of the pension systems in some countries and the design of provident fund pension systems. For instance, the study by de Souza et al. [7] concluded that ‘health does not present a barrier to raising the retirement age in Latin America, even in the longer term’. Moreover, if the CCT programs that target the elderly within the region continue and expand, it is pertinent to ask whether 65 is a meaningful institutional boundary for all countries and about the financial sustainability of such transfers with that age threshold. The 65 age threshold may be meaningful for Guyana or Suriname in 2020, but not necessarily for Mexico and Brazil, where the CCTs to the elderly have the largest coverage. For the latter, the resulting old-age threshold using our methodology is around 70 in both ages for the year 2020. In addition, the changes between 2020 and 2050 point to the need to constantly review the old-age threshold, the public policies associated with it, and the increases in life expectancy.

In Argentina, Chile, and Uruguay, spending on the population aged 65 and above accounts for around one-third of all contemporary public expenditure in health. In 2070, this figure will increase to 50% [41]. However, while this figure accurately represents the necessary increase in the number of people aged over 65, it does not take into account changes in their health status. A further benefit of the prospective measures we present here is that they reflect the widely agreed notion that healthcare expenditures are much more closely linked to proximity to death rather than chronological age [39]. As such, an old-age threshold based on the RLE, as suggested in this paper, offers a more refined tool to incorporate the demographic dynamics into the estimates of costs and health needs of older persons in Central and South America.

Such prospective measures as we have presented here can be more useful in (a) presenting a more holistic view of population aging which takes into account dynamic changes and differences in life expectancies, and (b) in exposing the potential rift between institutional frameworks based on chronological age and these changing population age structures. There are, however, certainly a number of limitations. The definition of a boundary to old age as RLE = 15 can certainly be described as arbitrary. Fixing this over time misses out a further degree of dynamic change over time in morbidity at older ages. Clearly, a more finely grained approach based on individual characteristics is preferable [46].

A further challenge relates to the reliability of past estimates of mortality and population, as well as the validity of future projections. The process of gathering accurate statistics over the past seventy years in some Central and Southern American countries has been more highly developed than others [47]. Without doubt, data issues will accompany some of the estimates presented above, and may well explain some of quirks in the past trends (such as the unusual shape of the old-age threshold calculated for Guyana (Fig 3) or the history of life expectancy in Belize (Fig 1). We have based our calculations on what is arguably the world’s most commonly utilized population (projection) dataset, namely the United Nations’ World Population Prospects [28]. There may be criticisms of the methodology used in various aspects of the UN estimation and projection framework which have been elucidated elsewhere [48] as well as alternative approaches to forecasting population [49–51] and using alternative variants [52]. The uncertainty associated with projections could be better demonstrated with probabilistic measures of aging (as was performed elsewhere) [40]; however the data to perform such a task were not made publicly available with the *Revision* used in this analysis.

Finally, a common challenge with any measure of ‘dependency’ is determining what these defined ‘groups’ (of ‘dependent’ and, by definition, ‘non-dependent’) actually represent and what ‘dependency’ actually means. An implicit assumption of the *OADR*, for example, is that the population aged 15/18/20-60/65 is the ‘working-age population’. Sometimes this can be falsely equated to the ‘labor force’. Each definition is problematic in its own, and needs to be reconsidered in the light of differentials in not only (age- and gender-specific) labor force participation rates but also the nature of the labor market itself (e.g. industrial base, formal/informal) [53]. Furthermore, the ways by which inter- and intra-generational transfers are made in different contexts is crucial to understanding the institutional challenges associated with aging. This has been shown to tremendous effect by the National Transfer Accounts framework generally [54] and for Central and South America [55].

Adopting a prospective measure of aging, then, simply allows for an alternative means of picturing changes in population structure through a simplistic measure [56]. While this may help communicate the reality of ‘demographic aging’, the real challenges are fundamentally linked to the creation and reform of institutions as well as broader social, economic, political and cultural issues. Aging in the region will occur in a context of prevailing inequality, persistent high levels of poverty and weak social security systems [57]. There are wide differences in the institutional building of social policies and the public or private provision of health, care and social security. Some countries started earlier and have a more universalistic approach (Uruguay, Argentina); others have a combination of a universal approach with a wide participation of the private sector (Chile); others limit the access to public social security and particular health services to workers in the formal sector and their families (Mexico and Brazil) and others have not been able to build public health institutions and provide social security to large sectors of the population (most Central American and Andean countries) [57]. In spite of the differences, most of these countries share a stratified access to health services with large variations in the quality of the services provided within the country. Some face the challenge of unsustainable pension systems that were designed last century at a moment where the population above 65 was very small. More recently, the region is moving between paradigms regarding social policies: between a stratified and privatized provision of health and a more universalist health system; from non-contributive pensions to individual capitalization; with a spread of CCT to the elderly (usually 65 and above) as a way to reduce poverty.

## Conclusion

While a tremendous human achievement, the process of population aging in Central and Southern America will undoubtedly bring a number of challenges to extant institutions. It will also require the development and growth of new and nascent institutions in a sustainable manner. On the other hand, from a theory of change perspective, there is still much work to do in order to meet the Sustainable Development Goals [58] as pertaining to older people, as well as the goals set out in the Madrid International Plan of Action on Aging [59]. A small part of this effort will revolve around how population aging itself is conceptualized and communicated. We argue that the prospective measures offer a helpful complement to existing measures as a means of communicating the dynamic changes in aging over time and differences over space.

## Supporting information

S1 Table Median age, prospective median age, proportion of population aged 65+, old-age threshold (age at which RLE = 15 years); proportion above old age threshold; old age dependency ratio and prospective old age dependency ratio, Latin America, 1980, 2020, 2050. Source: UN World Population Prospects 2019 Revision.
(DOCX)
