# Supplementary material for: Prospective measures of aging for Central and South America
Source: PLoS One. 2020 Jul 24;15(7):e0236280. doi: 10.1371/journal.pone.0236280 (PMC7380630; doi:10.1371/journal.pone.0236280)
Supplement: S1 Table — Source: UN World Population Prospects 2019 Revision. (DOCX) [file pone.0236280.s001.docx]

**SI Table 1: Median age, prospective median age, proportion of population aged 65+, old-age threshold (age at which RLE=15 years); proportion above old age threshold; old age dependency ratio and prospective old age dependency ratio, Latin America, 1980, 2020, 2050. Source: UN World Population Prospects 2019 Revision**

|  | **Median Age** | | | **Prospective Median Age** | | | **Proportion of population aged 65+** | | | **Old-age threshold** | | | **Proportion above old age threshold** | | | **Old age dependency ratio (65+/20-64)** | | | **Prospective old age dependency ratio** | | |
| --- | --- | --- | --- | --- | --- | --- | --- | --- | --- | --- | --- | --- | --- | --- | --- | --- | --- | --- | --- | --- | --- |
|  | ***1980*** | ***2020*** | ***2050*** | ***1980*** | ***2020*** | ***2050*** | ***1980*** | ***2020*** | ***2050*** | ***1980*** | ***2020*** | ***2050*** | ***1980*** | ***2020*** | ***2050*** | ***1980*** | ***2020*** | ***2050*** | ***1980*** | ***2020*** | ***2050*** |
| **Central America** |  |  |  |  |  |  |  |  |  |  |  |  |  |  |  |  |  |  |  |  |  |
| Belize | 16.29 | 25.46 | 36.22 | 17.19 | 24.36 | 31.43 | 0.04 | 0.05 | 0.13 | 65.56 | 70.32 | 72.25 | 0.04 | 0.03 | 0.07 | 0.12 | 0.09 | 0.21 | 0.12 | 0.06 | 0.11 |
| Costa Rica | 20.50 | 33.44 | 45.80 | 26.17 | 32.26 | 40.62 | 0.04 | 0.10 | 0.24 | 66.53 | 72.57 | 75.51 | 0.04 | 0.05 | 0.12 | 0.08 | 0.17 | 0.42 | 0.08 | 0.08 | 0.17 |
| El Salvador | 17.77 | 27.45 | 39.31 | 26.10 | 25.94 | 33.44 | 0.03 | 0.09 | 0.16 | 63.62 | 69.55 | 72.23 | 0.04 | 0.06 | 0.10 | 0.08 | 0.16 | 0.27 | 0.10 | 0.11 | 0.15 |
| Guatemala | 16.69 | 22.82 | 33.00 | 25.83 | 21.28 | 27.04 | 0.03 | 0.05 | 0.11 | 62.53 | 70.33 | 72.74 | 0.04 | 0.03 | 0.06 | 0.07 | 0.10 | 0.19 | 0.10 | 0.06 | 0.09 |
| Honduras | 16.10 | 24.32 | 35.99 | 24.82 | 23.19 | 31.22 | 0.03 | 0.05 | 0.13 | 64.77 | 70.74 | 72.87 | 0.04 | 0.03 | 0.07 | 0.09 | 0.09 | 0.21 | 0.09 | 0.05 | 0.10 |
| Mexico | 17.28 | 29.15 | 39.31 | 22.50 | 29.14 | 35.19 | 0.04 | 0.08 | 0.17 | 66.64 | 69.20 | 72.27 | 0.04 | 0.05 | 0.10 | 0.10 | 0.13 | 0.29 | 0.09 | 0.09 | 0.16 |
| Nicaragua | 16.29 | 26.45 | 37.04 | 23.89 | 24.96 | 31.13 | 0.03 | 0.06 | 0.15 | 63.14 | 69.76 | 72.73 | 0.03 | 0.04 | 0.08 | 0.07 | 0.10 | 0.25 | 0.09 | 0.06 | 0.12 |
| Panama | 19.07 | 29.67 | 37.54 | 24.49 | 28.40 | 32.69 | 0.04 | 0.09 | 0.18 | 66.94 | 73.62 | 75.70 | 0.04 | 0.04 | 0.09 | 0.10 | 0.15 | 0.32 | 0.08 | 0.07 | 0.13 |
| **South America** |  |  |  |  |  |  |  |  |  |  |  |  |  |  |  |  |  |  |  |  |  |
| Argentina | 27.14 | 31.49 | 38.15 | 31.49 | 30.56 | 33.46 | 0.08 | 0.11 | 0.17 | 64.77 | 69.33 | 72.21 | 0.08 | 0.08 | 0.10 | 0.15 | 0.20 | 0.30 | 0.16 | 0.14 | 0.16 |
| Bolivia (Plurinational State of) | 19.47 | 25.54 | 34.29 | 29.31 | 23.44 | 28.97 | 0.04 | 0.07 | 0.13 | 61.17 | 71.62 | 72.69 | 0.06 | 0.04 | 0.07 | 0.09 | 0.14 | 0.22 | 0.14 | 0.08 | 0.11 |
| Brazil | 20.32 | 33.50 | 45.06 | 27.13 | 31.89 | 39.09 | 0.04 | 0.10 | 0.23 | 62.89 | 70.83 | 73.82 | 0.05 | 0.06 | 0.12 | 0.08 | 0.16 | 0.40 | 0.10 | 0.09 | 0.18 |
| Chile | 22.82 | 35.33 | 46.08 | 30.96 | 34.48 | 40.85 | 0.05 | 0.12 | 0.25 | 65.10 | 71.98 | 75.28 | 0.05 | 0.07 | 0.13 | 0.10 | 0.20 | 0.45 | 0.10 | 0.10 | 0.20 |
| Colombia | 19.06 | 31.26 | 43.95 | 25.95 | 30.06 | 39.05 | 0.04 | 0.09 | 0.21 | 64.96 | 71.16 | 73.84 | 0.04 | 0.05 | 0.12 | 0.08 | 0.15 | 0.36 | 0.08 | 0.08 | 0.17 |
| Ecuador | 18.60 | 27.89 | 36.98 | 25.40 | 26.54 | 31.38 | 0.04 | 0.08 | 0.16 | 64.83 | 71.39 | 74.22 | 0.04 | 0.04 | 0.08 | 0.10 | 0.14 | 0.28 | 0.10 | 0.07 | 0.13 |
| French Guiana | 23.11 | 25.07 | 31.08 | 31.50 | 24.52 | 25.84 | 0.05 | 0.06 | 0.13 | 63.43 | 70.16 | 73.99 | 0.06 | 0.03 | 0.07 | 0.10 | 0.11 | 0.24 | 0.11 | 0.06 | 0.11 |
| Guyana | 18.14 | 26.53 | 35.63 | 22.78 | 25.85 | 32.82 | 0.04 | 0.07 | 0.15 | 60.82 | 69.62 | 70.19 | 0.05 | 0.04 | 0.11 | 0.09 | 0.13 | 0.26 | 0.13 | 0.08 | 0.17 |
| Paraguay | 18.29 | 26.27 | 35.28 | 21.66 | 25.26 | 31.96 | 0.04 | 0.07 | 0.13 | 64.24 | 68.94 | 70.44 | 0.04 | 0.05 | 0.08 | 0.09 | 0.12 | 0.22 | 0.10 | 0.08 | 0.13 |
| Peru | 18.41 | 30.97 | 39.35 | 25.85 | 28.92 | 33.09 | 0.04 | 0.09 | 0.19 | 62.83 | 70.13 | 73.57 | 0.05 | 0.06 | 0.10 | 0.08 | 0.15 | 0.33 | 0.11 | 0.09 | 0.16 |
| Suriname | 18.79 | 28.96 | 36.31 | 20.88 | 28.41 | 32.56 | 0.04 | 0.07 | 0.14 | 63.46 | 65.78 | 68.23 | 0.05 | 0.07 | 0.12 | 0.10 | 0.12 | 0.25 | 0.12 | 0.12 | 0.19 |
| Uruguay | 30.15 | 35.75 | 42.54 | 34.83 | 34.90 | 37.80 | 0.11 | 0.15 | 0.22 | 65.25 | 71.02 | 73.77 | 0.10 | 0.10 | 0.13 | 0.19 | 0.26 | 0.39 | 0.19 | 0.16 | 0.20 |
| Venezuela (Bolivarian Republic of) | 18.82 | 29.60 | 38.01 | 21.69 | 29.09 | 34.76 | 0.03 | 0.08 | 0.15 | 64.65 | 67.23 | 69.94 | 0.03 | 0.06 | 0.11 | 0.07 | 0.14 | 0.26 | 0.08 | 0.11 | 0.17 |
